# High-Performance Polymer-Based Membranes for CO_2_ Separation: Recent Advances and Perspectives

**DOI:** 10.3390/membranes16060189

**Published:** 2026-06-01

**Authors:** Huimin Ma, Xiaoxue Jiang, Nianwei Man, Jing Zhao

**Affiliations:** State Key Laboratory of Materials-Oriented Chemical Engineering, College of Chemical Engineering, Nanjing Tech University, Nanjing 211816, China; mahuimin@njtech.edu.cn (H.M.); xiaoxuejiang@njtech.edu.cn (X.J.); mannianwei-njtech.edu.cn@njtech.edu.cn (N.M.)

**Keywords:** polymer membrane, CO_2_ separation, conventional polymer membranes, microporous polymer membranes, mixed matrix membranes

## Abstract

The urgent demand for energy-efficient CO_2_ separation technologies has propelled significant advancements in polymer-based CO_2_ separation membranes over the past decade. This review systematically examines three primary classes of these membrane materials: conventional dense polymer membranes, microporous polymer membranes, and mixed matrix membranes (MMMs). We analyze their distinct transport mechanisms, advantages, and the persistent challenges of permeability-selectivity trade-offs, physical aging, and scalability that have hindered widespread industrial adoption despite significant laboratory advances. Furthermore, we offer a forward-looking perspective on critical research directions, including the molecular design of stable microporous polymers, the evolution of MMMs towards continuous hybrid architectures, and the development of scalable ultrathin membrane fabrication techniques. By integrating materials innovation with engineering practicality, polymer-based membranes are poised to play a transformative role in sustainable carbon management.

## 1. Introduction

The escalating concentration of atmospheric CO_2_ presents a formidable challenge to global climate stability and sustainable development [1,2]. Beyond its role in climate change, CO_2_ is also an impurity that must be removed from natural gas and biogas to meet pipeline specifications, prevent corrosion, and maintain calorific value [3]. Consequently, efficient CO_2_ separation technologies are critically needed for both carbon capture, utilization, and storage (CCUS) applications and natural gas sweetening [4,5]. The gas streams encountered in these contexts vary widely in composition, pressure, and temperature, ranging from dilute, low-pressure flue gas in post-combustion capture to high-pressure natural gas mixtures. This diversity necessitates a suite of adaptable separation technologies. Absorption using amine solvents is a mature and widely used technology; however, it suffers from high capital and operational costs, significant energy consumption during solvent regeneration, and environmental concerns related to solvent degradation and corrosion [6]. By comparison, membrane technology has garnered burgeoning interest. Membrane-based CO_2_ separation is an affordable and attractive technology due to its intrinsic advantages, including high energy efficiency, operational simplicity and modularity for easy scale-up, and a small environmental footprint [7]. With extensive process optimization, membrane systems are projected to meet ambitious cost targets for CO_2_ capture and natural gas upgrading, positioning them as a competitive technology for sustainable carbon management and energy production [8].

Membrane materials constitute the core of this technology, and their properties ultimately dictate both separation performance and commercial viability [9,10,11]. The landscape of membrane materials under investigation for CO_2_ separation is diverse, encompassing polymer-based membranes and inorganic membranes such as zeolites, carbon molecular sieves, metal-organic frameworks (MOFs), and covalent organic frameworks (COFs) [12,13,14,15,16,17,18]. While these inorganic or crystalline materials often exhibit exceptional thermal stability and molecular sieving capabilities that can surpass the Robeson upper bound, their widespread industrial adoption remains elusive [19,20]. This is primarily due to formidable challenges in scalable, defect-free fabrication, high capital costs and inherent brittleness, which complicate module assembly and long-term operation under process conditions. In this context, polymer-based membranes have emerged as the predominant platform for gas separation, commanding a central role in both academic research and industrial applications [21,22,23,24]. Their ascendancy is rooted in a suite of compelling advantages: superior film-forming ability, solution processability, mechanical flexibility, and, critically, the capacity for cost-effective, large-scale manufacturing. These attributes position polymeric membranes as the most promising solution to meet the immense scalability demands of CO_2_ separation, where membrane areas on the order of millions of square meters are required. Over the past decade, significant research efforts have been devoted to advancing these materials, from conventional dense polymers to microporous polymers and mixed matrix architectures to unlock higher efficiencies in CO_2_ separation. Despite these efforts, the deployment of polymer-based membranes is confronted with persistent scientific and engineering challenges that must be solved in future work. A fundamental limitation is the well-established trade-off between permeability and selectivity, encapsulated by the Robeson upper bound, which constrains the performance ceiling of pure polymeric materials. Beyond this intrinsic constraint, issues of long-term stability from CO_2_-induced plasticization and physical aging under realistic operating conditions pose significant hurdles [25].

**Figure 1:**
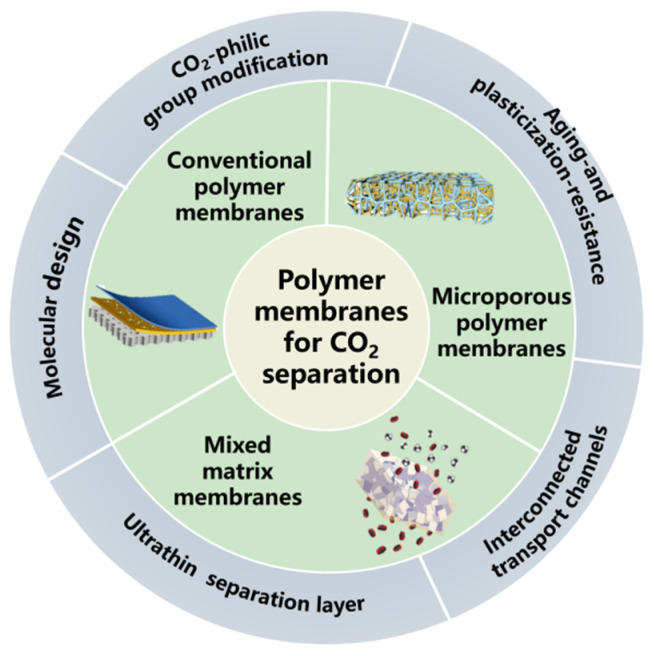
Schematic illustration of main research topics in polymer-based membranes for CO_2_ separation.

The scope of this review focuses on the recent development of advanced polymer-based membrane materials for CO_2_ separation in the past 5 years (Figure 1). We systematically examine three major classes of materials, namely conventional dense polymer membranes, microporous polymer membranes, and mixed matrix membranes (MMMs), highlighting their distinct transport mechanisms, recent breakthroughs, and persistent challenges. Moreover, we provide a forward-looking perspective on critical research directions essential for translating laboratory innovations into industrial reality. These include the molecular-level design of next–generation polymers to stabilize microporosity against aging, the evolution of MMMs toward structurally continuous hybrid architectures to eliminate interfacial defects, and the parallel advancement of ultrathin membrane engineering using scalable techniques such as interfacial polymerization to convert high permeability into practical permeance. By integrating fundamental materials innovation with engineering practicality, this review aims to offer a comprehensive roadmap for the continued development of polymer-based CO_2_ separation membranes as a cornerstone of sustainable carbon management.

## 2. Conventional Polymer Membranes

Traditional polymer membranes utilize linear or crosslinked organic polymers as the separation medium and achieve selective gas separation via the solution–diffusion mechanism or facilitated transport mechanism [26]. They represent the most promising class of CO_2_ separation membrane materials for industrial applications, offering distinct advantages such as excellent solvent processability, scalability, and low cost [27]. However, polymeric membranes are persistently challenged by the trade–off between permeability and selectivity–a fundamental constraint elucidated by the Robeson upper bound–as well as long-term stability issues including plasticization-induced swelling and physical aging.

Based on the characteristics of segmental thermal motion, polymeric membranes are broadly classified into rubbery and glassy polymers, which exhibit fundamentally distinct gas separation mechanisms. Rubbery polymers possess flexible backbone structures and high chain mobility, and their separation behavior is primarily governed by differences in gas solubility within the membrane, leading to preferential solubility selectivity for CO_2_. Representative materials include poly(ethylene oxide) (PEO, Merck KGaA, Darmstadt, Germany) and its copolymers [28,29]. In contrast, glassy polymers feature rigid molecular skeletons and restricted segmental motion. Their separation relies on the free volume elements formed by inefficient chain packing, which enable diffusivity selectivity for gas molecules of varying kinetic diameters [30]. Typical examples include polyimides (PI, Sigma-Aldrich, St. Louis, MO, USA), as well as polymers of intrinsic microporosity (PIM) and thermally rearranged polymers (TR-polymers), both of which exhibit enhanced free volume.

To transcend the limitations of the solution-diffusion mechanism, facilitated transport membranes introduce a reactive separation concept by incorporating carriers (e.g., amine groups) capable of reversible reactions with CO_2_ into the polymer matrix. These materials facilitate transmembrane transport through reversible chemical reactions between the carrier and CO_2_, demonstrating exceptionally high selectivity in low-pressure CO_2_/N_2_ separation scenarios. Polyvinylamine (PVAm, Lupamin™, BASF SE, Ludwigshafen, Germany) is a representative example of such materials [31].

In this section, we review the recent research advances and key challenges associated with traditional rubbery polymer membranes, glassy polymer membranes, and facilitated transport membranes. Glassy microporous polymers will be specifically discussed in the following section.

### 2.1. Rubbery Polymer Membranes

Rubbery polymers, characterized by their highly flexible backbones and strong chain segmental mobility, primarily rely on solubility selectivity to achieve component separation in gas separation processes [27]. Compared to glassy polymers, these materials exhibit superior film-forming properties, enabling the fabrication of thinner separation layers and offering potential for large-scale production, which lays the foundation for their industrial application. PEO–based rubbery polymers represent the most extensively studied and promising class of CO_2_/N_2_ separation membrane materials for industrial deployment. The ether oxygen units within these polymers can engage in Lewis acid-base interactions with CO_2_, enabling preferential CO_2_ adsorption and thereby achieving efficient separation. However, the practical application of these materials faces two major challenges. First, the plasticization effect under high-pressure conditions. Rubbery polymers, with their high chain mobility, are prone to plasticization-induced swelling in high-pressure CO_2_/CH_4_ separation scenarios, leading to increased interchain spacing and a significant decline in selectivity, which limits their applicability under high-pressure operating conditions [32]. Second, the inherent crystallization tendency of PEO–based polymers. Their regular chain structure tends to form dense chain packing, resulting in a sharp decrease in CO_2_ permeability and constraining the enhancement of separation performance [33].

To address the issues of high crystallinity and low permeability in PEO, current research has focused on optimization through molecular design and material blending, which can be mainly categorized into the following three strategies: (1) synthesis of copolymers containing PEO segments in the main or side chains [28]; (2) formation of crosslinked PEO networks through the reaction of PEO monomers to inhibit PEO crystallization [34]; (3) introduction of low-molecular-weight PEO into other polymers to form blend systems [35].

**Figure 2:**
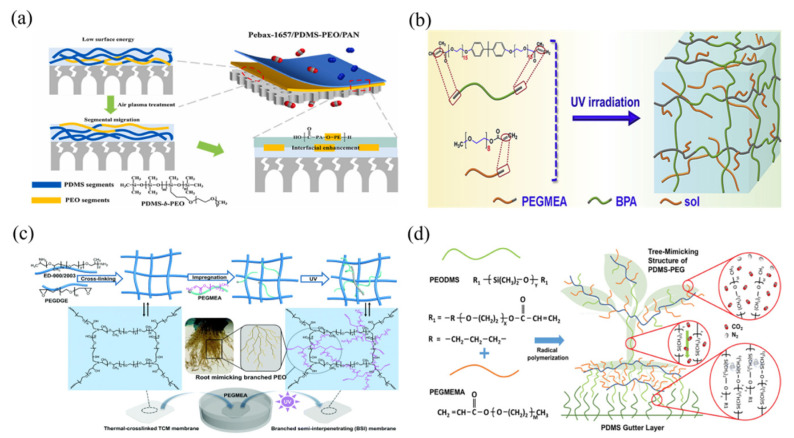
Schematic illustration of representative rubbery polymer membrane structures: (**a**) ultrathin Pebax composite membrane [40]; (**b**) crosslinked PEO formed via free radical polymerization [43]; (**c**) crosslinked PEO formed via epoxy ring-opening polymerization, and the branched semi-interpenetrating (BSI) membrane prepared via incorporating polyPEGMEA into crosslinked PEO [46]; (**d**) PEO-PDMS copolymer [52].

A representative commercial PEO–based block copolymer is Pebax^®^ (Arkema, Colombes, France), which comprises alternating rigid polyamide and flexible PEO segments. The PEO segments govern the preferential solubility of CO_2_, while the polyamide domains provide mechanical robustness [36]. Pebax^®^ exhibits a CO_2_ permeability of about 70–100 Barrer and a CO_2_/N_2_ selectivity of around 50 [37]. Although its permeability is moderate compared to other membrane materials, its outstanding solution processability allows for the fabrication of ultrathin composite membranes via scalable and industrially mature solution-coating methods. This renders it highly attractive for practical applications, and its performance limitations can be appropriately compensated for through strategies such as mixed matrix membrane design. Selyanchyn and Jiang et al. [38,39] proposed a plasma-assisted approach to increase the surface hydrophilicity of the gutter layer, enabling the defect-free coating of a Pebax selective layer with a thickness down to 30 nm. On this basis, Liu et al. [40] introduced an amphiphilic block copolymer, polydimethylsiloxane-block-polyethylene oxide (PDMS-b-PEO), into the PDMS(Sigma-Aldrich, St. Louis, MO, USA) gutter layer (Figure 2a). By leveraging the surface segregation of the PEO segments, this strategy effectively suppressed the formation of a dense SiO_x_ layer on the gutter layer surface during plasma treatment, while simultaneously enhancing interfacial compatibility with the Pebax separation layer and improving the structural stability of the membrane. The resulting membrane exhibited a CO_2_ permeance of 2142 GPU and a CO_2_/N_2_ selectivity of 36.

Crosslinked PEO is typically synthesized via ring-opening polymerization or free radical polymerization of reactive groups within PEO monomers [41,42]. Compared to PEO copolymer membranes, crosslinked PEO exhibits a higher content of ether oxygen groups and a completely amorphous structure, which generally endows it with a higher CO_2_ permeability [27]. For instance, the crosslinked PEO membrane formed via epoxy ring-opening polymerization of O,O′-Bis(2-aminopropyl) polypropylene glycol–block–polyethylene glycol–block–polypropylene glycol (ED-600, Huntsman, The Woodlands, TX, USA) and Poly (ethylene glycol) diglycidyl ether (PEO-500) exhibits a CO_2_ permeability of approximately 200 Barrer, while that prepared by UV-initiated free radical polymerization of Poly(ethylene glycol) diacrylate (PEGDA, Aladdin, Shanghai, China) and Polyethylene glycol methyl ether acrylate (PEGMEA, Sigma-Aldrich, St. Louis, MO, USA) achieves a CO_2_ permeability of around 500 Barrer. Sun et al. [43] employed bisphenol A ethoxylate diacrylate (BPA, Sigma-Aldrich, St. Louis, MO, USA), a monomer with a larger molecular size, in place of PEGDA in the free radical polymerization to increase the free volume within the membrane (Figure 2b). This modification resulted in a CO_2_ permeability of 1711 Barrer while maintaining a high CO_2_/N_2_ selectivity of 44.

Building upon the synthesis of PEO copolymers or crosslinked PEO, the further incorporation of low-molecular-weight PEG increases the density of ether oxygen units within the membrane, thereby enhancing preferential CO_2_ adsorption. Meanwhile, it functions as a plasticizer, modulating the free volume and optimizing molecular diffusion behavior, thus yielding substantial enhancements in both permeability and selectivity. Zhu et al. [44] developed a hybrid three-dimensional cross-linked scaffold by polymerizing acrylic inorganic acrylo polysilsesquioxane (POSS, Sigma-Aldrich, St. Louis, MO, USA) cage with PEGMEA, into which poly(ethylene glycol) (PEG, Sigma-Aldrich, St. Louis, MO, USA) or poly(ethylene glycol) dimethyl ether (PEGDME, Sigma-Aldrich, St. Louis, MO, USA) of varying molecular weights was introduced as a swelling agent. This approach enhanced the gas diffusivity in the membrane by nearly 9-fold, achieving a CO_2_ permeability as high as 5608 Barrer, which exceeds that of state-of-the-art membrane materials such as PIMs while maintaining a selectivity of around 40 and stable separation performance during long-term operation. Similarly, Sun et al. [45] incorporated PEGDME into a BPA-PEGMEA cross-linked network, increasing the CO_2_ permeability to 4883 Barrer. Although the incorporation of small molecules significantly enhances membrane performance, the associated structural instability–such as the leaching of small molecules under high pressure and the decline in mechanical strength–limits their practical application. To address this issue, Zhu and Jiang et al. [46,47] employed thermal- and photo-initiated polymerization methods to in situ synthesize poly-PEGMEA molecular brushes within Pebax or PEO matrices (Figure 2c). The abundant PEO side chains and terminal methyl groups in the molecular brushes facilitated CO_2_ adsorption and diffusion. Moreover, the extensive entanglements between poly-PEGMEA and Pebax ensured good mechanical stability even at high PEGMEA contents (up to 90 wt%). The membrane exhibited a CO_2_ permeability of 832 Barrer, with CO_2_/N_2_ and CO_2_/H_2_ selectivities of 63.5 and 20.8, respectively, at a feed pressure of 20 bar.

As outlined above, crosslinked PEO membranes are capable of achieving superior separation performance relative to commercial Pebax^®^ membranes. However, studies on their fabrication into composite membrane configurations remain relatively limited, particularly for crosslinked PEO prepared via free radical polymerization. This is primarily attributed to the difficulty in synthesizing soluble polymers suitable for such approaches, rendering the preparation of ultrathin composite membranes via industrially mature solution-coating methods a considerable challenge [48,49]. Zhang et al. [48] synthesized a high-molecular-weight, soluble crosslinked PEO via atom transfer radical polymerization (ATRP) and directly coated it onto a PDMS gutter layer, fabricating a defect-free thin-film composite (TFC) membrane with a thickness of 506 nm. The resulting membrane exhibited a CO_2_ permeance of 850 GPU and a CO_2_/N_2_ selectivity of 37. Building on this approach, a defect-free, amorphous PEO/C6 hybrid layer with a thickness of only 110 nm was subsequently constructed by depositing an adhesive polydopamine interlayer—which exhibits high affinity for PEO–onto the PDMS surface prior to coating with a PEO solution containing C6 molecules [49]. This optimized membrane achieved a CO_2_ permeance of 2200 GPU with a CO_2_/N_2_ selectivity of 27. In our previous work [50], ultrathin composite membranes based on crosslinked PEO formed via epoxy ring-opening polymerization delivered a CO_2_ permeance of up to 3200 GPU, with a CO_2_/N_2_ selectivity of approximately 23. These findings demonstrate that PEO-based polymers, when fabricated into ultrathin composite membranes, can achieve high CO_2_ permeance, often outperforming glassy polymers that are prone to physical aging.

PDMS is a typical rubbery polymer characterized by exceptionally high chain mobility, which confers ultrahigh CO_2_ permeability (~3800 Barrer). However, PDMS membranes exhibit relatively low CO_2_/N_2_ selectivity, falling short of the benchmark selectivity of >20 required for carbon capture applications. Recent studies have demonstrated that copolymerizing or blending PDMS with poly(ethylene oxide) (PEO) can synergistically integrate the advantages of both materials, enabling the fabrication of CO_2_ separation membranes that simultaneously achieve high permeability and high selectivity. In our previous work [51], a molecular-scale hybrid material (PEO-Si), comprising PEO segments as the organic component and siloxane as the inorganic part, was introduced into the PDMS matrix. The ether oxygen groups in the organic component enhance the CO_2_ affinity, thereby facilitating selective CO_2_ adsorption. Meanwhile, the siloxane units in the inorganic part undergo polycondensation to form higher molecular weight species, which improve the structural stability of the membrane through physical entanglement and interactions with the PDMS matrix. The incorporation of this molecular hybrid material increased the CO_2_/N_2_ selectivity of the PDMS membrane from 13.9 to 28.2, while maintaining a high CO_2_ permeance of 2860 GPU. Zhang et al. [52] developed a dendritic polymer network by copolymerizing PDMS and PEO monomers. In this architecture, hydrophobic PDMS brushes act as “roots”, ensuring firm attachment to the gutter layer; PDMS crosslinkers function as the “xylem”, facilitating rapid gas transport through the membrane; and hydrophilic PEO groups serve as “leaves”, selectively adsorbing CO_2_ molecules (Figure 2d). When this copolymer was coated onto hollow fiber supports, the resulting membrane exhibited a CO_2_ permeance of approximately 2700 GPU and a CO_2_/N_2_ selectivity of around 21. This method can produce hollow fiber composite membranes via a simple dip-coating process, easily enabling large-scale fabrication and the subsequent assembly into hollow fiber membrane modules, thus showing good application prospects.

Due to the limited modification strategies available for PDMS, research in PDMS-dominant membranes remains scarce. Currently, PDMS is primarily utilized for its high permeability as a gutter layer in ultrathin composite membranes [40]. It serves to provide a smooth and defect-free surface that facilitates the formation of an ultrathin separation layer, while preventing pore penetration of the coating solution, which could otherwise result in significant mass transfer resistance.

In summary, rubbery polymer membranes, particularly PEO-based membranes, have achieved impressive CO_2_ permeance, leveraging their excellent solution-processability and solubility selectivity. However, a critical assessment reveals three major issues that limit their applications. First, plasticization under high-pressure CO_2_ conditions remains inadequately addressed; most studies report performance at low pressures (<5 bar). Second, the industrial scalability of fabrication methods for crosslinked PEO and copolymer systems is not well established; many high-performance materials are synthesized via routes that are incompatible with continuous coating processes. For instance, plasma or UV/O_3_ equipment used for gutter layer modification can only process membrane materials in batch mode, thereby hindering the continuous preparation of large-area flat-sheet membranes, which is a prerequisite for assembly into spiral wound membrane modules. Therefore, for high-performance carbon capture membrane materials, it is still necessary to develop membrane fabrication methods that are compatible with the industrially mature continuous coating equipment. Last, future efforts must prioritize mixed-gas high-pressure testing, mechanical robustness assessment, and the development of scalable processing routes that preserve the exceptional transport properties demonstrated at the laboratory scale.

### 2.2. Glassy Polymer Membranes

Glassy polymers, with rigid backbones and restricted segmental mobility, primarily govern gas separation through diffusivity selectivity, enabling molecular sieving of gas molecules with distinct kinetic diameters [53,54]. The free volume elements arising from rigid chain packing preferentially allow the permeation of smaller CO_2_ molecules while hindering the diffusion of larger CH_4_ molecules, thereby achieving efficient CO_2_/CH_4_ separation. Compared with rubbery polymers, glassy materials exhibit superior mechanical strength and pressure resistance, enabling them to withstand high-pressure operating conditions [55]. This renders them particularly advantageous in high-pressure CO_2_/CH_4_ separation scenarios such as natural gas upgrading, establishing them as the most intensively studied and industrially promising membrane materials in the field of CO_2_/CH_4_ separation. Taking PI as an illustrative example, conventional PIs such as Matrimid^®^ have already achieved industrial adoption by virtue of their excellent mechanical properties, thermal stability, pressure resistance, and relatively low cost, while exhibiting high selectivity in CO_2_/CH_4_ separation.

However, the practical deployment of glassy polymers is confronted with three fundamental challenges: First, dense chain packing generally results in relatively low CO_2_ permeability [56]; Second, they are inherently constrained by the permeability-selectivity trade-off [54]; and Third, they are susceptible to physical aging and CO_2_-induced plasticization during prolonged operation, compromising membrane stability under high-pressure conditions [57]. To address the aforementioned issues, current efforts focus on molecular structure design and material engineering. Molecular design that incorporates rigid backbones strategically disrupts polymer chain packing, generating microporous architectures with enhanced free volume and thereby promoting superior gas permeability [58,59]. Representative materials include thermally rearranged polymers (TR-polymers) and polymers of intrinsic microporosity (PIMs), which are currently research hot spots in the field of glassy polymer membranes. These two categories of materials will be specifically introduced in the section on “Microporous Polymer Membranes.” In addition to rigidity adjustment, functionalizing with polar groups [60,61], covalent cross-linking [62,63], and constructing MMMs are also important approaches to enhancing the performance of glassy polymer membranes [64,65]. These modulation strategies are consistent with those used for microporous polymer membranes and will also be introduced later in the subsequent content.

### 2.3. Facilitated Transport Membranes

**Figure 3:**
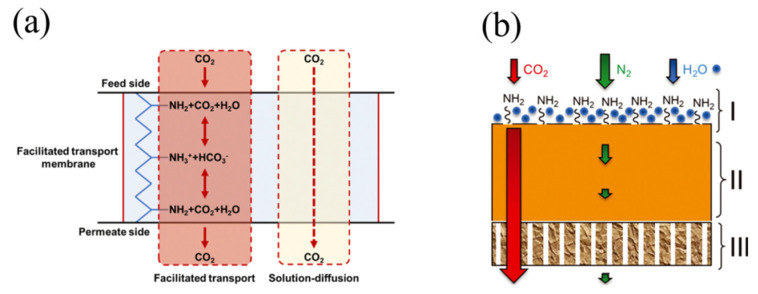
(**a**) Schematic illustration of CO_2_ transport through facilitated transport membranes [76]; (**b**) Illustration of the underlying mechanism behind hybrid-integrated (HI) membranes [23].

Facilitated transport membranes operate based on the principle of reactive separation, wherein carriers capable of reversible chemical reactions with CO_2_ are introduced into the polymer matrix to achieve selective CO_2_ transport across the membrane [66]. Amine is the most commonly used CO_2_ carrier, exhibiting acid-base affinity with CO_2_ molecules. Its transport mechanism is illustrated in Figure 3. Under humidified conditions, amine carriers function as solid-state bases, reversibly reacting with CO_2_ and water to form bicarbonate ions. On the downstream side of the membrane, bicarbonate ions are converted back to CO_2_, which desorbs from the membrane. Meanwhile, the transport of N_2_ molecules relies solely on the solution-diffusion mechanism, thereby endowing the membrane with high CO_2_/N_2_ selectivity. In low-pressure CO_2_/N_2_ separation scenarios such as flue gas carbon capture, facilitated transport membranes represent the most intensively studied class of materials. Currently, PVAm stands as the most representative membrane material for CO_2_ separation via facilitated transport. Owing to its abundant primary amine groups, excellent film-forming properties, and superior CO_2_-facilitated transport capability, PVAm has been extensively studied and exhibits significant potential for commercialization [67,68]. To achieve higher-efficiency CO_2_ separation, current research efforts on facilitated transport membranes focus on the following strategies: increasing carrier density, introducing mobile carriers, constructing ultrathin composite membranes [23], and incorporating porous fillers to prepare mixed matrix membranes [69,70].

Li et al. [71] synthesized PVAm-based copolymer membrane materials enriched with multiple functional groups, including primary amino groups, carbonate ions, and quaternary ammonium cations, to enhance carrier types and carrier content. The introduction of multiple carriers overcomes the limitations associated with excessively high content of a single carrier, while synergistic effects among different groups enable more efficient CO_2_ adsorption and transport within the membrane material. Employing the above copolymer as the separation layer, a composite membrane was prepared by a facile coating method, achieving an excellent CO_2_ permeance of 1842 GPU and a CO_2_/N_2_ selectivity of 160. Sandru and coworkers proposed grafting polyamine onto a high-permeability polymer matrix to form a molecularly thin surface layer rich in primary amine groups, achieving highly selective enrichment of CO_2_ from the feed gas. By combining this surface-facilitated transport layer with the underlying high-permeability polymer, the membrane exhibited ultrahigh selectivity (up to over 1000) in CO_2_/N_2_ separation while maintaining a high CO_2_ permeability of 1200 Barrer. Targeting industrial applications, Sheng et al. [72] developed a scaled-up fabrication process for multilayer composite membranes comprising PVAm-dominant separation layers, demonstrating high and stable CO_2_/N_2_ separation performance. Although facilitated transport membranes exhibit excellent CO_2_ separation performance, their separation behavior is highly sensitive to humidity conditions. For instance, Zhang et al. [73] developed a high-performance PVAm-based composite membrane for CO_2_ separation from oilfield-associated gas. At 25 °C, when the feed-side relative humidity was increased from 40% to 100%, the CO_2_/CH_4_ selectivity increased from 22 to 62, while the CO_2_ permeance increased by a factor of approximately four. Belaissaoui et al. [74] prepared a hybrid fixed-site carrier facilitated transport membrane based on polyallylamine matrix. At 50 °C, when the relative humidity on the feed side was increased from 20% to 90%, both CO_2_ and N_2_ permeance of the membrane increased, with CO_2_ permeance showing an exponential increase (from 75 GPU to 300 GPU), reflecting the significant promoting effect of increased water content on the CO_2_-amine carrier reaction and CO_2_ transport process.

Through simulations and calculations, Li et al. [75] discovered that constructing a strongly polar environment, reducing the nitrogen atom spacing of amino groups, and building abundant CO_2_ transport channels may reduce the humidity dependence of the reactivity selectivity mechanism. In addition to humidity, impurity gases in the feed gas, such as oxygen and trace amounts of SO_2_, significantly affect the facilitated transport performance of the amine carrier. Wang et al. [70] investigated the operational stability of the PVAm-based mixed matrix membrane under simulated flue gas conditions (4.5 vol% CO_2_, 6.5 vol% O_2_, 140 ppm SO_2_, balance N_2_). With only a trace amount of SO_2_ in the feed gas, the preferential reaction between SO_2_ and the membrane’s amine groups inhibited CO_2_ transport, leading to a decrease in CO_2_ permeance from 3092 GPU to 2100 GPU, and CO_2_/N_2_ selectivity from 184 to 130, respectively. However, when O_2_ and SO_2_ were removed from the feed gas, the membrane separation performance quickly recovered to its initial level, demonstrating good reversibility and poisoning resistance.

Overall, facilitated transport membranes represent one of the most selective platforms for CO_2_/N_2_ separation under low-pressure, humidified conditions. However, their practical deployment is constrained by several critical issues. Humidity dependence is the most fundamental limitation: while high selectivity is achieved under fully humidified conditions, real flue gas streams in membrane modules exhibit significant humidity fluctuations that would cause significant performance variability. More critically, the long-term chemical stability of amine carriers under realistic operating conditions including exposure to oxygen, trace SO_x_, NO_x_, and temperature cycles has not been systematically studied. The potential for carrier deactivation under variable feed compositions poses a risk for industrial applications. Furthermore, the fabrication of ultrathin facilitated transport membranes with defect-free selective layers becomes increasingly challenging as carrier content increases, creating a tension between selectivity enhancement and mechanical integrity. Future research must move beyond idealized humidity-controlled tests and address carrier stability, multi-component feed tolerance, and scalable membrane formation under industrially relevant conditions.

## 3. Microporous Polymer Membranes

Microporous polymers represent a class of glassy polymeric materials characterized by rigid, contorted molecular structures that inhibit efficient chain packing, thereby giving rise to intrinsic microporosity [77,78]. The core design philosophy of these materials is to transcend the inherent limitations of conventional glassy polymers–namely, dense chain packing and limited free volume–by engineering interconnected and stable microporous transport pathways while preserving the benefits of a rigid backbone. Through molecular engineering that modulates conformational rigidity and interchain packing behavior, microporous polymers can attain substantially higher free volume and gas permeability than traditional glassy polymers, while maintaining high mechanical strength and thermal stability [79,80]. Consequently, microporous polymers are recognized as one of the next-generation membrane materials for CO_2_ separation and represent a vibrant research frontier within the field of polymeric membranes. The structural characteristics of microporous polymers underpinning their excellent performance mainly include three aspects. First, the rigid and contorted molecular backbones (e.g., spirocyclic structures, triptycene units) effectively disrupt ordered chain packing, generating abundant intrinsic micropores with dimensions ranging from 0.5 to 2 nm that serve as rapid diffusion pathways for gas molecules. Second, the interconnected nature of these micropores establishes a three-dimensional transport network throughout the membrane, enabling CO_2_ permeabilities that exceed those of conventional glassy polymers by one to three orders of magnitude. Third, the inherent rigidity of the backbone confers exceptional resistance to plasticization and high-pressure conditions, endowing these materials with superior long-term operational stability compared to traditional glassy polymers in high-pressure CO_2_/CH_4_ separation applications [81,82].

**Figure 4:**
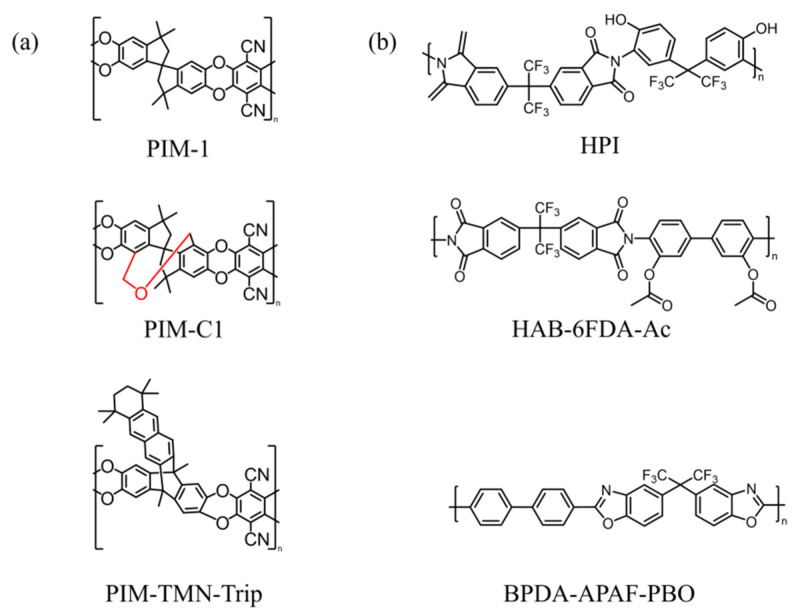
Schematic structures of typical microporous polymers: (**a**) PIMs; (**b**) TR-polymers. Cyclic-locked PIM-1 (PIM-C1), Tetramethyl tetrahydro naphthalene (TMN), triptycene (Trip), High-Performance Polyimides (HPIs), 3,3′-dihydroxy-4,4′-diamino-biphenyl (HAB), 2,2′-bis-(3,4-dicarboxyphenyl) hexafluoropropane di-anhydride (6FDA), 4,4′-(Hexafluoroisopropylidene)bis(2-aminophenol) (APAF), 4,4′-biphenyltetracarboxylic dianhydride (BPDA), Polyb-enzoxazoles (PBOs).

Based on their molecular structure and pore formation mechanism, microporous polymers are primarily classified into two categories: Polymers of Intrinsic Microporosity (PIMs) and Thermally Rearranged Polymers (TR-polymers). PIMs refer to a class of polymeric materials with permanent micropores. Their microporosity does not rely on external templates or fillers. Instead, it stems from the rigid, contorted, and asymmetric backbones of the polymer chains, which prevent efficient chain packing and result in continuous, permanently existing micropores in the solid state (Figure 4a) [80,83]. TR-polymers are functional membrane materials obtained through high-temperature thermal treatment, during which precursor polymers are transformed into aromatic polybenzoxazole or polypyrrolone structures. This conversion generates high free volume and microporous architectures (Figure 4b) [84,85].

Although microporous polymers exhibit impressive separation performance, their practical application remains hindered by a fundamental challenge: the long-term stability of the microporous structure. Owing to the inherent non-equilibrium nature of glassy polymers, microporous polymers are susceptible to physical aging during operation, leading to free volume decay, micropore collapse, and a consequent decline in gas permeability over time [86]. Furthermore, the excessive pursuit of high free volume often compromises size-sieving precision [87]; when the micropore size distribution is overly broad, achieving accurate molecular discrimination for gas pairs such as CO_2_/CH_4_ and CO_2_/N_2_ becomes difficult, resulting in limited selectivity. In addition, the relatively complex synthesis routes and high production cost of microporous polymers pose further constraints on their large-scale industrial adoption [88]. To address these challenges, current research efforts focus on molecular design, chain architecture engineering, and membrane configuration optimization, encompassing the following key strategies.

### 3.1. Precise Engineering of Microporous Structure

Through rational molecular design, the degree of contortion of the polymer backbone and the interchain interactions of polymers can be finely tuned to narrow the micropore size distribution while maintaining high free volume, thereby enhancing the size-sieving capability for CO_2_. Typical approaches include the incorporation of bridging units with tailored rigidity, functionalization of substituents on spirocyclic structures, and copolymerization of rigid monomers with flexible spacer segments [89,90,91].

**Figure 5:**
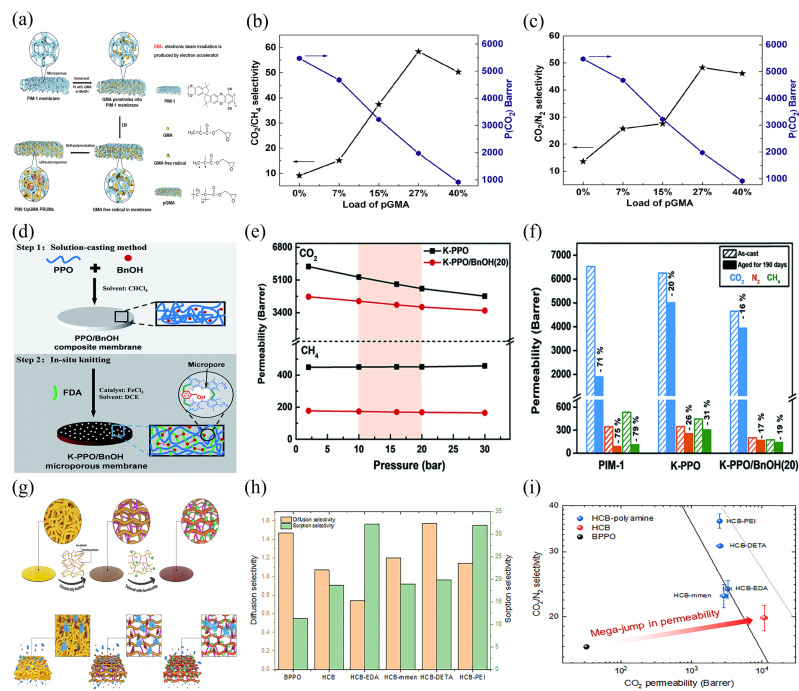
Structure construction strategies and gas separation performance of microporous polymer membranes (**a**–**c**) PIM–1/pGMA–x polyolefin reweaved ultramicroporous membranes [93]: (**a**) Schematic illustration of the formation of polyolefin reweaved ultramicroporous membranes; (**b**) CH_4_ permeability and CO_2_/CH_4_ selectivity; (**c**) CO_2_ permeability and CO_2_/N_2_ selectivity. (**d**–**f**) In situ knitted microporous polymer membrane (KMPM) system [102]: (**d**) Schematic illustration of the synthesis of KMPMs; (**e**) CO_2_ and CH_4_ permeabilities of K–PPO and K–PPO/BnOH(20) membranes as a function of pressure; (**f**) Effect of aging time on the gas permeabilities of PIM–1, K–PPO, and K–PPO/BnOH(20) membranes. (**g**–**i**) HCP/BPPO-based chemically driven molecular sieve membranes [103]: (**g**) Schematic illustration of membrane structure evolution and mass transport pathways in HCP membranes; (**h**) Evolution of separation performance of BPPO, HCB, and HCB-amine membranes during successive post-treatment steps; (**i**) Influence of diffusivity selectivity and solubility selectivity on their separation performance.

Lai et al. [25] synthesized microporous ladder polymers via the catalytic arene-norbornene annulation (CANAL) method by introducing bridging units of varying rigidity and modulating the substituent groups on the spirocyclic structure. Fluorene or dihydrophenanthrene units were incorporated into the monomer design, endowing the polymer backbone with a three-dimensional distorted structure. This architecture enhances interchain twisting and restricts chain packing, thereby achieving high fractional free volume while narrowing the micropore size distribution, which significantly improves the size-sieving capability for CO_2_. The aged membrane exhibited a CO_2_/CH_4_ selectivity of 46 and a CO_2_ permeability of 600 Barrer in mixed-gas tests, along with an H_2_/CH_4_ selectivity as high as 186. Zhang et al. [92] introduced rigid triptycene units into thermally rearranged (TR) polymers to achieve synergistic control of backbone distortion and interchain interactions. This strategy enabled precise modulation of the micropore size distribution and reinforced size-sieving performance. The membrane thermally rearranged at 400 °C showed an H_2_/CH_4_ selectivity of 83.6 and an H_2_ permeability of 345.3 Barrer, representing increases of 78% and 34%, respectively, compared to the non-TR precursor. Chen et al. [93] proposed a strategy for fabricating polyolefin reweaved ultra-micropore membrane (PRUM) (Figure 5a). In this approach, olefin monomers are uniformly dispersed into a pristine membrane (e.g., PIM-1) via solution-based diffusion, and copolymerization design incorporating rigid monomers and flexible segments enables the formation of tunable microporous channels. By adjusting the loading of the olefin polymer, the pore size can be precisely controlled to achieve efficient gas separation. The resulting membranes exhibited a CO_2_ permeability of 1976 Barrer with CO_2_/CH_4_ and CO_2_/N_2_ selectivities reaching 58.4 and 48.3, respectively. This rigid-flexible copolymerization strategy offers a versatile and efficient route to fabricate sub-nanometer pore-sized gas separation membranes with broad applicability.

### 3.2. Mitigation of Physical Aging

Chemical crosslinking [94,95,96], thermal annealing post-treatment [97,98], or incorporation of nanofillers can enhance interchain constraints [99,100,101], thereby suppressing chain relaxation and free volume decay. These approaches not only delay physical aging but also simultaneously improve plasticization resistance under high-pressure conditions.

Niu et al. [62] introduced a carboxyl-containing phenolphthalin-based diamine, 2-(bis(3-amino-4-hydroxyphenyl)methyl) benzoic acid (AHPBA, Sigma-Aldrich, St. Louis, MO, USA), into the synthesis of 4,4′-Hexafluoroisopropylidene diphthalic anhydride-2,4,6-trimethyl-1,3-phenylenedimin (6FDA-DAM, Tianjin Zhongtai Materials Technology Co., Ltd., Xiqing District, Tianjin, China) polyimide for copolymerization. The carboxyl groups in AHPBA served as reactive sites for crosslinking the synthesized polyimide with ethylene glycol. Covalent crosslinking significantly enhanced the membrane’s resistance to plasticization and physical aging, with no obvious plasticization observed under 30 atm CO_2_ and stable performance maintained after 60 days of aging, highlighting its excellent potential for long-term operation. Wu et al. [102] synthesized polyphenylene oxide/benzyl alcohol (PPO/BnOH) membranes via an in situ knitting approach, incorporating formaldehyde dimethylacetal as a crosslinker to construct a highly crosslinked microporous network while simultaneously integrating hydroxyl functional units into the polymer skeleton to enhance CO_2_ affinity (Figure 5b). By tailoring the crosslinking density, both the pore size and pore chemistry of the membranes were finely modulated. The resulting membranes exhibited an ultrahigh CO_2_ permeability of 4651 Barrer, a CO_2_/CH_4_ selectivity of 27, as well as excellent resistance to plasticization (up to 30 bar) and long-term physical aging (over 190 days).

### 3.3. Affinity Enhancement Through Functionalization

Introducing polar groups (such as carboxyl, amine, or fluorine-containing groups) or other CO_2_-philic sites into microporous polymer skeletons can enhance dipole-quadrupole interactions or Lewis acid-base interactions with CO_2_. This strategy improves solubility selectivity without compromising high permeability, thereby optimizing separation performance for CO_2_/CH_4_ and CO_2_/N_2_.

Rodriguez et al. [104] synthesized six polymers of intrinsic microporosity (PIMs) featuring the same benzodioxane backbone but diverse functional groups (such as nitrile, carboxylic acid, amine, tert-butoxycarbonyl, and so on). Their work aimed to elucidate the structure-property relationships among polymer chemistry, CO_2_ sorption affinity, and competitive sorption effects in mixed-gas separation. Notably, the amine-functionalized PIM-1 (PIM– NH_2_) exhibited the highest CO_2_/CH_4_ sorption selectivity. Correspondingly, in mixed-gas permeation tests, PIM–NH_2_ demonstrated unprecedented performance enhancements: CO_2_/CH_4_ and CO_2_/N_2_ mixed-gas permselectivities increased by 140% and 250%, respectively, compared to pure-gas tests. Moreover, due to enhanced chain rigidity from interchain hydrogen bonding, PIM–NH_2_ retained excellent CO_2_/CH_4_ mixed-gas selectivity (>20) even at pressures up to 26 atm, indicating superior plasticization resistance. Lee et al. [103] constructed membranes with permanent microporosity to enable rapid gas diffusion and molecular sieving. Subsequently, four different polyamines were introduced into the pores to fine-tune both the pore architecture and CO_2_–membrane interactions (Figure 5c). Their findings revealed that larger alkyl amines functionalized the larger pore regions while preserving ultra-micropores, resulting in an increase in solubility selectivity from 19 to 32 and achieving optimal CO_2_/N_2_ separation performance. Fan et al. [105] incorporated a fluorinated-cardobased diamine (FFDA, Tianjin Zhongtai Materials Technology Co., Ltd., Xiqing District, Tianjin, China) into polybenzoxazole (PBO, Sigma-Aldrich, St. Louis, MO, USA) structures to prepare TR membranes with enhanced gas separation performance. The cardo groups improved the polymer’s molecular sieving capability through interchain hydrogen bonding and π–π stacking, while the aromatic fluorine atoms significantly boosted CO_2_ sorption. Following thermal rearrangement, the membranes developed pores of approximately 3 Å in diameter, accelerating the diffusion of CO_2_ and H_2_. Compared to their precursor membranes, the modified TR membranes exhibited a 227.3% increase in CO_2_ permeability and a 37.5% improvement in CO_2_/CH_4_ selectivity.

In summary, microporous polymers have dramatically enhanced the CO_2_ separation performance of polymer-based membranes, with PIMs and TR-polymers achieving CO_2_ permeabilities surpassing conventional glassy polymers by orders of magnitude. However, the field faces a critical juncture: the very structural features (rigid, contorted backbones) that confer high permeability also lead to key limitations of physical aging, processing difficulty, and often broad pore size distributions that compromise molecular sieving ability. It is extremely challenging to achieve high and stable CO_2_ permeance using microporous polymer thin films. While molecular design strategies have made impressive strides in narrowing pore size distributions and introducing CO_2_-philic functionalities, these approaches typically increase synthetic complexity and cost, raising questions about their economic viability. Moreover, the vast majority of reported performance metrics are derived from pure-gas tests on thick, self-standing films; data on mixed-gas performance under realistic pressures, long-term stability under practical conditions, and module-level performance remain scarce. The translation of microporous polymers from laboratory curiosities to industrially relevant membrane materials will require a paradigm shift from simply maximizing free volume to achieving a balanced combination of high permeability, stable microporosity over multi-year timescales, and compatibility with scalable ultrathin film fabrication processes.

## 4. Mixed Matrix Membranes

Mixed matrix membranes represent a class of hybrid membranes fabricated by incorporating porous fillers into a polymer matrix. Their core design concept is to synergistically combine the solution processability of polymers with the molecular sieving capability of porous fillers, aiming to overcome the inherent Robeson upper bound that limits conventional polymeric membranes [106]. Through rational selection of the polymer matrix, porous filler, and interfacial engineering strategy, MMMs can simultaneously achieve high permeability, high selectivity, and high stability while maintaining good film-forming properties. As such, MMMs are regarded as one of the most promising membrane candidates for next-generation applications and represent a focal point of current research. From a performance perspective, the synergistic effects of MMMs are manifested on three levels. First, the intrinsic pore channels of porous fillers provide rapid transport pathways for CO_2_, substantially enhancing the CO_2_ permeability [107,108]. Second, the molecular sieving characteristics or CO_2_-affinity sites of the fillers contribute to improved selectivity [109,110]. Third, the anchoring effect of fillers on polymer chains restricts chain mobility, endowing MMMs with superior plasticization resistance and anti-physical aging performance compared to the pristine polymer matrix [100,111,112].

Despite their promise, the scalable implementation of MMMs continues to be hindered by a critical challenge: the interfacial compatibility between the filler and the polymer matrix. Poor interfacial compatibility may lead to two detrimental outcomes: (i) the formation of non-selective voids at the interface, which can induce Knudsen diffusion or even viscous flow, leading to a drastic loss of selectivity [113]; (ii) excessively strong interfacial interactions may lead to pore blockage by polymer chains, negating the expected permeability enhancement [114]. Furthermore, the density of fillers is typically higher than that of polymers, making them prone to sedimentation during membrane casting and solvent evaporation. This can lead to non-ideal structures such as uneven filler distribution and local agglomeration, further compromising membrane performance [113]. To address these challenges, current research efforts are directed toward interfacial engineering, structural regulation, and membrane configuration optimization, encompassing the following four key strategies.

### 4.1. Surface Functionalization of Fillers

By introducing functional groups compatible with the polymer matrix onto the filler surface, interfacial interactions can be finely tuned by enhancing non-covalent interactions or forming covalent bonds. This strategy eliminates non-selective defects while preserving the open pore channels of the fillers, thereby achieving an ideal interfacial structure. Xiang et al. [115] reported the in situ incorporation of 2-aminobenzimidazole during the synthesis of ZIF-7 to prepare amino-functionalized ZIF-7, which enhanced interfacial compatibility with the PEO matrix. The resulting non-covalent interfacial interactions improved membrane selectivity to 55, with a CO_2_ permeability of 215 Barrer. Covalent bonds between fillers and the matrix can be formed under ambient conditions through reactions such as epoxy-amine chemistry or radical polymerization [116,117,118]. Jiang et al. [117] introduced two UiO-66-type fillers–amino-functionalized UiO-66-NH_2_ and isopropenyl-functionalized UiO-66-MA–into a crosslinked PEO matrix to optimize CO_2_ capture performance. During UV-induced copolymerization, the reactive UiO-66-MA underwent in situ covalent crosslinking with methyl acrylate-terminated PEO, forming efficient gas transport pathways and excellent interfacial adhesion. In contrast, membranes incorporating non-reactive UiO-66-NH_2_ exhibited relatively inferior interfacial morphology. The resulting membrane containing UiO-66-MA demonstrated a high gas permeability of 1450 Barrer with a CO_2_/N_2_ selectivity of approximately 45.8.

### 4.2. Construction of Three-Dimensional Continuous Filler Network

**Figure 6:**
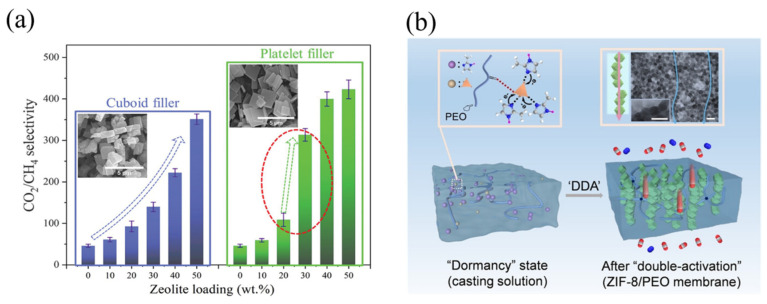
High-loading mixed matrix membranes (**a**,**b**): (**a**) The selectivity difference between cuboid-shaped and platelet-shaped Na-SSZ-39 MMMs. (Inset) SEM images show the morphology difference of two zeolites [119]; (**b**) Dormancy and double-activation strategy for preparation of MMMs with high ZIF-8 loadings [123].

Increasing the filler content beyond the percolation threshold enables the formation of a continuous, interconnected network within the membrane. Under such conditions, the transport behavior becomes dominated by the filler phase, thereby maximizing its molecular sieving potential and transcending the intrinsic permeability-selectivity trade-off of the polymer matrix. Tan et al. [119] homogeneously dispersed high-aspect-ratio Na-SSZ-39 zeolites–featuring both excellent CO_2_ affinity and well-defined molecular-sieving channels–into a Matrimid polyimide matrix at high loadings, followed by thermal annealing to form defect-free polymer-zeolite interfaces. When the loading reached 30–50 wt%, a sharp increase in membrane performance was observed (Figure 6a), indicating the formation of a percolating gas permeation highway across the membrane. The resulting membrane exhibited a CO_2_/CH_4_ selectivity exceeding 420 and a CO_2_ permeability as high as 8280 Barrer, surpassing those of state-of-the-art polymer membranes and even most zeolite membranes reported to date. Furthermore, the membrane demonstrated outstanding long-term stability (aging resistance), with negligible performance decay after one year.

In situ synthesis methods, owing to their ability to promote filler dispersion and interfacial adhesion, have been widely adopted for fabricating high-loading MOF-based MMMs [120,121,122]. Li et al. [123] proposed a “dormancy and double-activation” (DDA) strategy to prepare MMMs with a high MOF loading of 55 wt% (Figure 6b). The high concentration of MOF precursors suppressed crystallization in the casting solution, enabling molecular-level homogeneous mixing and uniform embedding within the polymer matrix. A subsequent two-step activation process induced MOF formation: alkali treatment promoted nucleation to generate small-sized porous nanocrystals, while excess ligands activated metal ions to enhance conversion efficiency. The interconnected MOF nanocrystals formed quasi-continuous transport pathways, substantially improving gas permeability. The optimized MMM achieved a CO_2_ permeability of 2841 Barrer—approximately five times higher than that of the pristine polymer membrane–along with a CO_2_/N_2_ selectivity of 36.

### 4.3. Development of Ultrathin MMMs Composite Membranes

Coating an ultrathin MMMs separation layer onto a porous substrate can reduce mass transfer resistance, significantly enhancing CO_2_ permeance, thereby increasing membrane processing capacity. However, ultrathin mixed-matrix separation layers are more susceptible to interfacial defects, imposing stricter requirements on filler particle size, dispersibility, and matrix-filler interfacial interactions. Ideally, fillers should be nanoscale or even sub-10 nm in size, and surface modification is often necessary to achieve superior interfacial compatibility and strong interfacial interactions.

**Figure 7:**
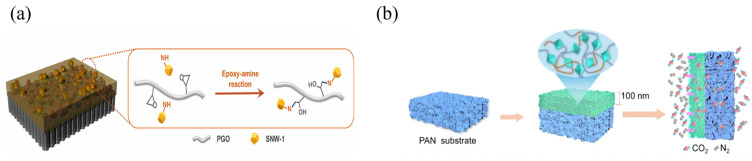
Ultrathin mixed matrix membranes: (**a**) Schematic illustration of the network structure of PGS-series MMMs [116]; (**b**) Schematic of the preparation and coating of PEO-MOF layer membranes [118].

Wang et al. [70] synthesized Schiff base network-1 (SNW-1) nanoparticles with a size of 40 nm, covalently grafted PVAm onto the filler surface, and subsequently incorporated the modified fillers into a PVAm matrix to fabricate mixed-matrix membranes. The polymer modification improved filler dispersion and interfacial compatibility, enabling the formation of defect-free separation layers with a thickness of only 120 nm. The resulting ultrathin mixed-matrix membrane exhibited a CO_2_ permeance of 3092 GPU and a CO_2_/N_2_ selectivity of 184. This approach is simple, cost-effective, and holds great promise for large-scale applications. Yeong et al. [116] integrated SNW-1 with a flexible rubbery comb copolymer, poly(glycidyl methacrylate-co-poly(oxyethylene methacrylate)) (PGO) (Figure 7a). The excellent adhesive properties of PGO, combined with the stable covalent bonds between SNW-1 and PGO via epoxy-amine reactions, significantly enhanced filler-polymer interfacial compatibility. This optimization facilitated the fabrication of defect-free ultrathin separation layers with a thickness of merely 60 nm. The resulting membranes demonstrated outstanding gas separation performance: at an optimal SNW-1 loading of 20 wt%, the CO_2_ permeance reached 3241 GPU with a CO_2_/N_2_ selectivity of 32.1. Fan et al. [118] proposed a reverse synthesis strategy, wherein PEO monomers were copolymerized with vinyl-functionalized UiO-66 nanoparticles to construct a polymer-MOF network (Figure 7b). This approach enabled the formation of ultrathin, defect-free separation layers with thicknesses below 100 nm, achieving a high MOF loading of 40 wt% within the poly(ethylene oxide) matrix. Under simulated mixed-gas conditions with 83% relative humidity, the membrane achieved a CO_2_ permeance of 2793 GPU and a CO_2_/N_2_ selectivity of 21.6.

### 4.4. Screening of Novel Functional Fillers

**Figure 8:**
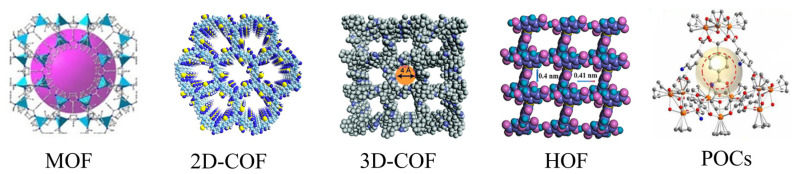
Schematic diagram of the pore structure of emerging porous materials [124,125,126].

In addition to traditional zeolites, emerging porous materials such as MOFs, covalent organic frameworks (COFs), hydrogen-bonded organic frameworks (HOFs), and porous organic cages (POCs) have become prominent fillers in current MMMs research due to their tunable pore structures and abundant chemical functional sites (Figure 8). Their high specific surface area, strong CO_2_ affinity, and favorable compatibility with polymer matrices offer more options for overcoming interfacial compatibility challenges.

MOFs are porous crystalline materials formed through the self-assembly of metal ions or clusters with organic ligands via coordination bonds [127,128]. Characterized by highly tunable pore structures and abundant chemical functional sites, MOFs are currently among the most extensively studied fillers. Li et al. [129] developed phase-separated mixed-matrix membranes (PS-MMMs) by blending two immiscible polyimides, wherein MOF particles were selectively incorporated into one polymer phase. When the localized packing density exceeded 50 vol%, a percolation network was achieved at a MOF loading of only 19 wt%. The resulting PS-MMM exhibited a CO_2_ permeability of 1385 Barrer with a CO_2_/N_2_ selectivity of 15. Lee et al. [130] proposed a polymer-MOF (polyMOF) system constructed using carboxylated PIM-1 (cPIM-1) as the ligand. This intrinsically microporous ligand coordinated with metal ions to form polyMOF nanoparticles of approximately 100 nm in size. Compared to control MOFs, these polyMOFs combined the pore architectures of both MOFs and PIM-1, exhibiting enhanced ultra-microporosity conducive to efficient molecular sieving. Concurrently, cPIM-1 served as a surface modifier, endowing the polyMOFs with improved compatibility with the PIM-1 matrix and enhanced dispersibility in the casting solution. The resulting polyMOF/PIM-1 MMM demonstrated exceptional CO_2_ separation performance, achieving a CO_2_ permeability of 9659 Barrer and a CO_2_/N_2_ selectivity of 21.5.

HOFs are a class of crystalline porous materials formed through the self-assembly driven by intermolecular hydrogen bonds, characterized by mild synthesis conditions, recyclability, and tunable pore structures [131,132]. Wang et al. [133] designed and synthesized a unique metal–hydrogen-bonded organic framework, HOF-21, which was processed into nanofillers using amine-based modulators to achieve uniform dispersion within the Pebax matrix. Benefiting from its hybrid bonded framework structure, HOF-21 exhibits a suitable pore size of approximately 0.35 nm and demonstrates excellent structural stability under humid feed gas conditions. The synergistic effect of its multiple binding sites and continuous hydrogen-bonded network effectively facilitates CO_2_ transport. The resulting HOF-21 mixed-matrix membrane exhibits a CO_2_ permeability exceeding 750 Barrer, with a CO_2_/CH_4_ selectivity of approximately 40 and a CO_2_/N_2_ selectivity of approximately 60.

POCs are discrete porous molecules formed by cage units connected through rigid organic frameworks, featuring controllable pore sizes and good solubility that facilitate uniform dispersion within polymer matrices [134,135]. Guan et al. [126] synthesized a series of novel metal-organic cages (MOCs) with precise pore sizes between the molecular sizes of CO_2_ and N_2_/CH_4_. These MOCs achieved homogeneous dispersion in PIM-1. Notably, the phenyl-functionalized cage (MOC-Ph) significantly modulated polymer chain packing and optimized the membrane’s microporous structure, resulting in exceptional performance with a CO_2_ permeability up to 8803.4 Barrer and a CO_2_/N_2_ selectivity of 59.9.

In addition to the emerging porous crystalline materials mentioned above, carbon nanomaterials also represent an important type of filler in mixed matrix membrane research. Typical examples include carbon nanotubes (CNTs) [136], graphene oxide (GO) [137], and its reduced form (rGO) [138]. These materials feature high aspect ratios, excellent mechanical properties, and tunable one- or two-dimensional mass transport channel structures. Among them, CNTs can form quasi-continuous fast diffusion channels within the polymer matrix, significantly reducing gas transport resistance [139]. Meanwhile, GO and rGO, owing to their layered structures and abundant oxygen-containing functional groups, not only enable molecular sieving through interlayer spacing regulation but also improve compatibility with the polymer matrix via interfacial interactions [138]. Shen et al. utilized the molecular interactions between GO and Pebax to induce the ordered stacking of GO nanosheets, forming well-defined laminar GO structures. The confined interlayer spaces served as molecular sieving channels, enabling highly efficient selective separation with a CO_2_/N_2_ selectivity of 91 [140]. By adjusting the lateral size of GO nanosheets, the length of the gas transport pathways and the polymer chain mobility could be tuned [141]. Building on this, Yang et al. prepared an ultrathin mixed-matrix separation layer of Pebax/GO, achieving a CO_2_ permeance of 400 GPU and a CO_2_/N_2_ selectivity of 72 [142].

Overall, mixed matrix membranes represent a powerful approach to overcoming the intrinsic permeability–selectivity trade-off of polymers, yet the field has reached a point where the incremental improvements offered by filler incorporation must be critically weighed against the associated increases in complexity, cost, and processing difficulty. While interfacial engineering has emerged as the central pathway in MMM development, no single strategy universally resolves the filler–matrix compatibility challenge, as each approach carries inherent trade-offs. Surface functionalization, while effective at eliminating non-selective voids, often risks pore blockage, particularly when functional groups are excessively bulky. High-loading strategies that achieve filler percolation can indeed create 3D continuous transport highways, but the mechanical properties of such membranes (particularly their brittleness and adhesion to porous supports) are underexplored, even though these properties are critical for scalable fabrication. In situ synthesis methods offer excellent dispersion but could introduce batch-to-batch variability that challenges membrane uniformity and reproducibility. Furthermore, the cost and scalability of novel filler materials (MOFs, COFs, HOFs, POCs) are rarely discussed; many of these materials are synthesized via multistep routes using expensive precursors or solvents, often making them much more costly than the polymer matrices. The literature is replete with examples of MMMs that surpass the Robeson upper bound under idealized conditions, but systematic studies addressing long-term filler–matrix interface stability under realistic conditions (mixed-gas feeds, high-pressure operation, and temperature cycling) remain conspicuously absent. Additionally, the translation of MMMs into ultrathin composite membrane configurations, essential for achieving high permeance, remains a significant technical hurdle, as the presence of fillers exacerbates defect formation during thin-film coating. Without a realistic assessment of filler cost, synthesis scalability, and the trade-offs associated with each interface engineering strategy, the transition of MMMs from academic demonstrations to industrial products will remain elusive. Future research should therefore prioritize not only the interface engineering and discovery of novel filler materials but also the development of robust, scalable fabrication processes and rigorous long-term stability testing under industrially relevant conditions, which can bridge the gap between laboratory-scale breakthroughs and the industry deployment of membrane technologies for CO_2_ separations.

## 5. Conclusions and Perspectives

### 5.1. Conclusions

The past decade has witnessed substantial progress in the development of high-performance polymer-based membranes for CO_2_ separation, driven by the urgent need for energy-efficient CO_2_ separation technologies. This review has systematically examined three major classes of polymer-based membranes including conventional dense polymer membranes, microporous polymer membranes, and mixed matrix membranes, each of them offering distinct advantages and facing unique challenges in the quest to overcome the inherent trade-off effect, stability issues, and scalability challenges.

Conventional dense polymer membranes, including rubbery and glassy polymers, remain the most industrially mature platforms for CO_2_ separation. Rubbery membranes based on PEO can achieve high solubility selectivity and possess exceptional thin-film processability, particularly advantageous for post-combustion CO_2_/N_2_ separation. However, their practical deployment is constrained by high crystallinity that limits CO_2_ permeability and by plasticization under high-pressure CO_2_ conditions. Recent advances in copolymer design, crosslinking strategies, and ultrathin film fabrication have partially mitigated these limitations, with PEO-based ultrathin membranes achieving attractive CO_2_ permeance exceeding 2000 GPU. Glassy polymer membranes, exemplified by polyimides, have good diffusion selectivity to separate CO_2_ from larger gases such as CH_4_, and meanwhile offering superior mechanical strength and pressure resistance essential for natural gas upgrading. Nevertheless, conventional glassy polymers face the persistent challenge of limited free volume and susceptibility to physical aging, particularly when TFC membranes are used. Facilitated transport membranes achieve exceptional CO_2_/N_2_ selectivity under humid conditions by leveraging reversible CO_2_-amine chemistry. Despite their promise for low-pressure flue gas applications, these membranes remain critically dependent on feed humidity and vulnerable to carrier oxidation and saturation under high-pressure or oxygen-containing environments. Microporous polymer membranes, such as PIMs and TR-polymers, have expanded the performance envelope of polymers. By utilizing rigid, contorted molecular backbones that frustrate chain packing, these membranes generate interconnected pores enabling CO_2_ permeability much higher than conventional glassy polymers while maintaining excellent resistance to plasticization. However, the non-equilibrium nature of microporous polymers renders them highly susceptible to physical aging, which remains a critical barrier to their industrial application.

MMMs represent an attractive platform that synergistically combines the facile processability of polymers with the exceptional and highly tunable transport properties of fillers. This integration enables MMMs to achieve superior separation performance that surpasses the Robeson upper bound for polymers. Over the past five years, the field has witnessed rapid progress, highlighted by breakthroughs in the fabrication of high-loading MMMs, wherein the filler phase forms interconnected percolation pathways throughout the membrane. Such advancements have been driven by the development of novel filler materials and innovative membrane processing techniques, including thermal-annealing-based defect healing and solid-solvent processing methods. Despite these strides, persistent challenges such as interfacial incompatibility between the filler and polymer matrix, along with filler sedimentation, remain critical hurdles. Addressing these issues necessitates the continued advancement of sophisticated interface engineering strategies.

### 5.2. Perspective

Looking forward, the field of polymer-based CO_2_ separation membranes stands at an exciting point where fundamental materials innovation must converge with engineering practicality. Several strategic directions emerge as critical for translating laboratory membrane breakthroughs into industrial applications.

First, molecular-level design of next-generation polymer backbones must address the dual imperatives of high permeability and long-term stability. For microporous polymers, the challenge lies not merely in maximizing free volume but in stabilizing microporosity against physical aging over industrially relevant conditions and timescales. Approaches combining rigid contorted structures with controlled crosslinking or chain rigidification through post-synthetic modifications offer promising pathways. The incorporation of CO_2_-philic functionalities, such as polar groups, fluorinated moieties, or amine carriers, within microporous frameworks can enhance solubility selectivity without compromising microporosity.

Second, the evolution of MMMs toward structurally continuous hybrid materials represents a paradigm shift from simple filler-in-polymer composites to truly integrated systems. The strategy of “molecularly mixed composite membranes,” where discrete porous molecules such as organic cages are molecularly dispersed within polymer matrices, eliminates the traditional filler-matrix interface and its associated defects. Similarly, polyMOFs constructed from polymer ligands blur the distinction between polymer and filler, offering enhanced compatibility and processability. The development of 3D connected filler networks, achieved through ultrahigh filler loadings exceeding the percolation threshold, can establish continuous gas transport highways that bypass the polymer matrix entirely. These approaches demand new materials design but promise to overcome the fundamental limitations of conventional MMMs. Moreover, the exploration of emerging porous materials as fillers continues to expand the design space. Beyond MOFs, COFs, HOFs and POCs offer unique advantages, including solution processability and compatibility, bringing distinct characteristics to MMM design.

Third, ultrathin membrane engineering must advance in parallel with materials discovery to realize the full potential of high-permeability materials. The translation of intrinsic permeability (Barrer) to practical permeance (GPU) requires defect-free selective layers of about 100 nm thickness, a formidable challenge for mechanically fragile microporous polymers and high-loading MMMs. Recent innovations in solid-solvent processing have enabled the fabrication of ultrathin MMMs with filler loadings up to 80 vol% and thickness below 100 nm, achieving gas-sieving performance orders of magnitude higher than conventional polymers. Scalable fabrication techniques, including continuous coating, interfacial polymerization, and layer-by-layer assembly, must be adapted to these advanced materials to bridge the gap between bench-scale demonstrations and industrial module production. Among fabrication techniques, interfacial polymerization stands out for its unique capability to produce ultrathin microporous membranes. This method is particularly well-suited for microporous polymers with high chain rigidity, which are otherwise difficult to process into ultrathin defect-free films. Interfacial polymerization not only facilitates the formation of sub-100 nm selective layers but is also readily scalable for industrial production. Even in the presence of significant physical aging, the ultrathin nature of these membranes ensures sustained high gas flux, making this approach highly attractive for practical applications. It should be noteworthy that long-term stability of the ultrathin film structures under industry-relevant conditions (pressure cycling, temperature fluctuations, and feed impurities) has not been established. Scalable fabrication techniques must be evaluated not only on their ability to produce thin films but also on their ability to maintain structural integrity over the multi-year lifetime required for industrial operation in the future studies. Besides, process intensification through membrane systems is of critical importance to reduce the energy consumption and cost of the separation process. Recent studies have demonstrated that intelligent process design, rather than membrane performance alone, is crucial for making membrane-based CO_2_ separation commercially viable [143,144]. Readers seeking a more detailed treatment of membrane process intensification are directed to the excellent review by Fu et al. [145].

Fourth, although the primary focus of this Review is on microporous polymers and mixed matrix membranes, conventional dense polymer membranes, including both rubbery and glassy polymers, remain the workhorse of industrial gas separations, owing to their cost-effectiveness, high solution processability, and established manufacturing scale-up routes. For these materials, future developments should focus on four directions: (i) engineering of polymer structure via blending or copolymerization to enhance separation performance while maintaining processability; (ii) the use of chemical crosslinking strategies to suppress CO_2_-induced plasticization and physical aging, thereby improving long-term operational stability; (iii) making ultrathin selective layer to maximize their CO_2_ permeance; and (iv) blending these dense polymers with functional nanofillers as discussed above. The parallel pursuit of these strategic directions is essential for bridging the gap between laboratory-scale materials innovation and industrial-scale module production.

Fifth, a multiscale understanding of transport phenomena and aging mechanisms must underpin rational materials design. Advanced characterization techniques, including positron annihilation lifetime spectroscopy (PALS), high-resolution electron microscopy, and in situ spectroscopic methods, coupled with molecular simulation and machine learning, can elucidate the evolution of free volume, the nature of filler-polymer interfaces, and the kinetics of physical aging. Such insights are essential for developing predictive models that accelerate materials discovery and optimization.

In conclusion, the field of polymer-based CO_2_ separation membranes is entering an era of unprecedented opportunity, where molecular-level materials design, advanced characterization, and scalable manufacturing are converging to overcome historical limitations. The synergistic integration of microporous polymers, functional fillers, and sophisticated interface engineering is yielding membranes that transcend the Robeson upper bound and approach the performance of purely inorganic sieves while retaining the processability of polymers. Realizing the full potential of these advanced materials will require sustained interdisciplinary collaboration spanning polymer chemistry, materials science, chemical engineering, and process systems engineering, with the ultimate goal of deploying membrane technology as a cornerstone of sustainable carbon management.

Last, to achieve truly sustainable CO_2_ separation, future research must also address the environmental footprint of membrane fabrication. The adoption of green solvents and solvent-free processes is emerging as a critical, albeit often overlooked, pathway alongside performance enhancement. Beyond fabrication, the end-of-life recycling of membrane materials, especially given the large volume of plastic waste, are equally important for closing the material loop. We advocate that reporting of high-performance membranes should increasingly include assessments of solvent toxicity, recyclability, and energy consumption of the fabrication process. The convergence of advanced materials engineering with green solvent chemistry and low-energy manufacturing will be essential to sustainable CO_2_ capture technologies.

## Data Availability

No new data were created or analyzed in this study. Data sharing is not applicable to this article.

## Abbreviations

The following abbreviations are used in this manuscript:

PEO	Poly(ethylene oxide)
PI	Polyimides
PIM	Polymers of intrinsic microporosity
TR-polymers	Thermally rearranged polymers
PVAm	Polyvinylamine
PDMS-b-PEO	Polydimethylsiloxane-block-polyethylene oxide
ED-600	O,O′-Bis(2-aminopropyl) polypropylene glycol–block–polyethylene glycol–block–polypropylene glycol
PEO-500	Poly (ethylene glycol) diglycidyl ether
BSI	Branched semi-interpenetrating
PEGDA	Poly(ethylene glycol) diacrylate
PEGMEA	Polyethylene glycol methyl ether acrylate
BPA	Bisphenol A ethoxylate diacrylate
POSS	Inorganic acrylo polysilsesquioxane cage
PEG	Poly(ethylene glycol)
PEGDME	Poly(ethylene glycol) dimethyl ether
HI	Hybrid-integrated
ATRP	Atom transfer radical polymerization
TFC	Thin-film composite
PIM-C1	Cyclic-locked PIM-1
TMN	Tetramethyl tetrahydro naphthalene
HPIs	High-Performance Polyimides
HAB	3,3′-dihydroxy-4,4′-diamino-biphenyl
6FDA	2,2′-bis-(3,4-dicarboxyphenyl) hexafluoropropane dianhydride
APAF	4,4′-(Hexafluoroisopropylidene)bis(2-aminophenol)
BPDA	4,4′-biphenyltetracarboxylic dianhydride
TR-PBOs	Thermally Rearranged Polyb-enzoxazoles
CANAL	Catalytic arene-norbornene annulation
PRUM	Polyolefin reweaved ultra-micropore membrane
AHPBA	2-(bis(3-amino-4-hydroxyphenyl)methyl) benzoic acid
6FDA-DAM	4,4′-Hexafluoroisopropylidene diphthalic anhydride-2,4,6-trimethyl-1,3-phenylenedi-min
PPO	Polyphenylene oxide
BnOH	Renzyl alcohol
KMPM	Knitted microporous polymer membrane
HCPs	Hyper-crosslinked Polymers
FFDA	Fluorinated-cardobased diamine
PBO	Polybenzoxazole
DDA	Dormancy and double-activation
SNW-1	Schiff base network-1
PGO	Poly(glycidyl methacrylate-co-poly(oxyethylene methacrylate))
MOF	Metal-organic frameworks
COF	Covalent organic frameworks
HOFs	Hydrogen-bonded organic frameworks
POCs	Porous organic cages
PS-MMMs	Phase-separated mixed-matrix membranes
cPIM-1	Carboxylated PIM-1
polyMOF	Polymer-MOF
MOCs	Metal-organic cages
MOC-Ph	Phenyl-functionalized cage
